# Using Google Location History data to quantify fine-scale human mobility

**DOI:** 10.1186/s12942-018-0150-z

**Published:** 2018-07-27

**Authors:** Nick Warren Ruktanonchai, Corrine Warren Ruktanonchai, Jessica Rhona Floyd, Andrew J. Tatem

**Affiliations:** 1 WorldPop Project, Geography and Environment, University of Southampton, Southampton, SO17 1BJ UK; 2 Flowminder Foundation, Roslagsgatan 17, 11355 Stockholm, Sweden

**Keywords:** Human mobility, Mobile phone data, GPS tracker data

## Abstract

**Background:**

Human mobility is fundamental to understanding global issues in the health and social sciences such as disease spread and displacements from disasters and conflicts. Detailed mobility data across spatial and temporal scales are difficult to collect, however, with movements varying from short, repeated movements to work or school, to rare migratory movements across national borders. While typical sources of mobility data such as travel history surveys and GPS tracker data can inform different typologies of movement, almost no source of readily obtainable data can address all types of movement at once.

**Methods:**

Here, we collect Google Location History (GLH) data and examine it as a novel source of information that could link fine scale mobility with rare, long distance and international trips, as it uniquely spans large temporal scales with high spatial granularity. These data are passively collected by Android smartphones, which reach increasingly broad audiences, becoming the most common operating system for accessing the Internet worldwide in 2017. We validate GLH data against GPS tracker data collected from Android users in the United Kingdom to assess the feasibility of using GLH data to inform human movement.

**Results:**

We find that GLH data span very long temporal periods (over a year on average in our sample), are spatially equivalent to GPS tracker data within 100 m, and capture more international movement than survey data. We also find GLH data avoid compliance concerns seen with GPS trackers and bias in self-reported travel, as GLH is passively collected. We discuss some settings where GLH data could provide novel insights, including infrastructure planning, infectious disease control, and response to catastrophic events, and discuss advantages and disadvantages of using GLH data to inform human mobility patterns.

**Conclusions:**

GLH data are a greatly underutilized and novel dataset for understanding human movement. While biases exist in populations with GLH data, Android phones are becoming the first and only device purchased to access the Internet and various web services in many middle and lower income settings, making these data increasingly appropriate for a wide range of scientific questions.

**Electronic supplementary material:**

The online version of this article (10.1186/s12942-018-0150-z) contains supplementary material, which is available to authorized users.

## Background

Understanding human mobility and how it manifests across temporal and spatial scales is important across the health and social sciences [1], as mobility patterns drive important spatial processes from infrastructure and land use to infectious disease spread [2]. The health sciences have increasingly focused on human movement in recent decades, accounting for the importance of geographical context in driving health inequalities and exposure to environmental risks [3]. Geographical context is strongly linked to the critical concept of “neighbourhood” [3], or the spatial context of a given individual. Within the social sciences, this temporally dynamic concept of incorporating an individual’s experiences is foundational to informing how social inequalities persist through mechanisms such as racial segregation, how individuals are exposed to environmental hazards, and how accessibility varies to social and health resources [4]. Traditionally, studies examining geographical context have used the characteristics of the administrative unit that individuals reside within to quantify their exposure to risks or accessibility to various rather than an emergent understanding of exposure [3]. This ignores individual-level spatial and temporal variation in where people spend time [5], however, potentially smoothing over the unique mobility patterns of marginalized populations and subgroups.

More recently, these issues have been addressed using the concept of an individual’s activity space (defined as encompassing all the locations a person interacts with over time) [6, 7], yielding a much more accurate picture of risk and social context than residence alone. Along these lines, recent studies have found that using place of residence rather than actual activity space underestimated exposure to spatial risks by 16 and 7% in Vancouver and Southern California respectively [8]. Further, using an individualized understanding of activity space can uncover sources of social patterns and inequalities that would not be observed using a static, administrative-boundary-based understanding of neighbourhood, such as accessibility to healthcare services [9, 10], personal exposure to spatial risks [11], and social networks [12]. In particular, populations that are highly segregated will have strongly disparate activity spaces [13], which will cause geographically close groups of people to experience dramatically different realities.

Utilizing such activity-based approaches in the health and social sciences, however, requires a precise and broad understanding of geographical context and environmental exposure across time [14]. Because locations for certain activities are often very close in space (for example, work and commercial activity), data used to inform activity space should be ideally be spatially refined enough to enable identification of different location types [14]. These data should also be temporally broad enough to capture regular behaviour patterns across long periods with sufficient certainty [14]. Though various disciplines have explored how activity spaces over weekly and monthly periods affect transit and exposure to frequently visited areas such as physical activity spaces, schools, workplaces, and otherwise [6], the extent of exposures experienced over a more broad timescale such as years and decades have been less explored. This owes partly to lacking data on long-term mobility patterns at sufficient spatial resolutions, and remains a critical gap in our understanding of exposure to risks that lead to spatial outcomes such as cancer, obesity, and various inequalities that arise from long-term differences in accessibility between populations.

**Fig. 1:**
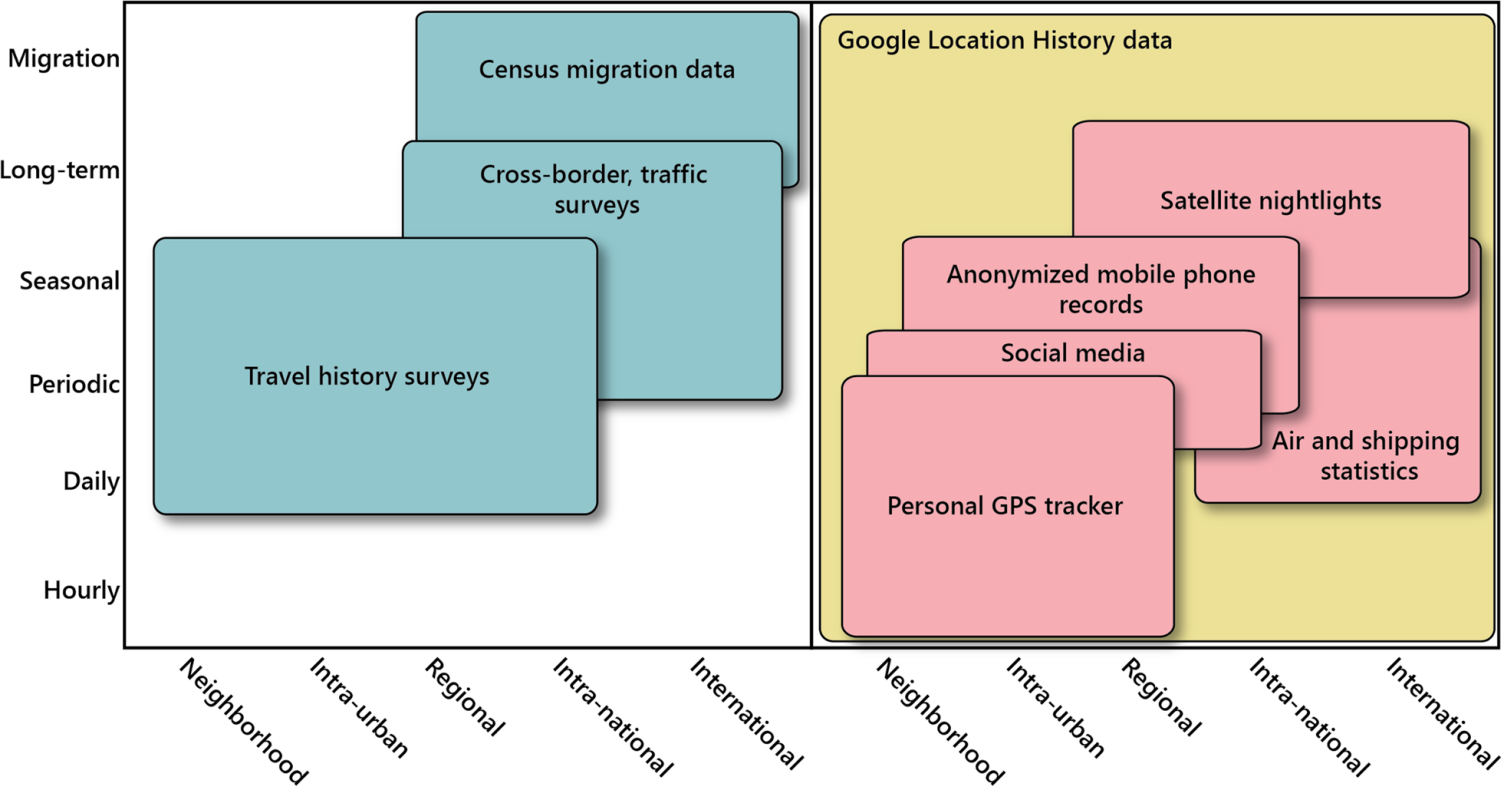
The information niche that Google Location History occupies. Adapted from [9]; left includes traditional mobility data, right includes mobility data available with more recent technologies. Google Location History data (yellow) record location points similarly to GPS trackers, while spanning timescales similar to mobile phone data, and cover a breadth of time spans and spatial scales not possible in other datasets

With recent technological advancements, a number of data sources on human movement have been used to inform activity space across temporal scales [15, 16] (Fig. 1). Traditionally, travel diaries have been an invaluable source of mobility information to inform activity spaces [13], as respondents can identify the specific locations used for various activities, which can then be identified in the context of the respondent’s residence. While data from personal GPS trackers provide information on short-distance, circulatory movement and can directly inform activity spaces [17], census-derived and population stock data inform longer-distance migratory movement, and exposure over longer periods [18]. Other data inform mobility at intermediate spatial and temporal scales, such as remotely sensed night-time light data that help infer where people are within cities over the course of a year [16, 19, 20], or social media data, which record the location where various social media services are used [21]. In some countries, data from mobile phones (call data records, or CDRs) provide national-scale coverage, recording the cell tower that calls and texts are routed through and the associated times over months or years [11, 22].

These sources of mobility data can also be significantly biased or have other drawbacks. Travel diaries are laborious to collect, for example, and subject to recall bias, especially when requesting the respondent to recall beyond several months [23]. Further, while CDRs have facilitated a national-level understanding of activity space and mobility, these data remain particularly difficult to obtain and use at present, however, requiring onerous data-sharing agreements with mobile operators, are treated as proprietary due to privacy concerns, and are spatially coarse as towers can be many kilometres apart in rural areas, and cannot typically track international movement. Social media and CDR data collection can also be highly biased, only recording location when calls and texts occur, or when social media services are used, causing CDRs to underestimate total travel distance and movement entropy [24].

Because of these drawbacks in current data, and a broader need to understand activity spaces across temporal scales, novel data are needed that can be easily collected with social and demographic information, cover long time periods, and identify locations of travel with high spatial precision. We explore here Google Location History (GLH) data as an underused source of human mobility information that could fill this niche in numerous research contexts. These data consist of geographic coordinates routinely recorded by Android phones, and are associated with a consolidated user account, allowing for location data that are recorded across all mobile devices that an individual has owned. GLH data have been collected in an opt-out, passive fashion for Android users since location services have been fully integrated into Android in 2012 [25]. Each user can quickly and freely access their own data through a web browser. In studies that use GLH data, users can download their associated data and provide it to researchers during surveys that include an appropriate informed consent process. Because location is identified using a combination of the phone’s internal GPS and connected WiFi devices and cell towers, we show that these data are as spatially refined as GPS tracker data while spanning years (Fig. 1). Further, the passively-collected nature of GLH data avoids many known biases from compliance issues in studies that use GPS trackers, and avoids recall bias found in self-reported travel history data.

Though potentially biased towards wealthier populations, GLH data are available from an increasingly large proportion of the world, as the Android user base has increased dramatically since 2012 [26], reaching over 1.4 billion active devices in 2015 [27]. In particular, these devices are popular as an affordable way to access the Internet in low and middle income settings [27], and worldwide, Android market share for accessing the Internet has surpassed Microsoft Windows [26].

As they have only become recently available, GLH data have not previously been used to understand patterns of human mobility in social science research. Therefore, critical questions must be addressed before they can be used to examine important issues in the social sciences. Here, we conducted a pilot study among Android users in the United Kingdom to address: (1) what proportion of Android users have GLH data enabled, and whether this correlates with use of various Google services; (2) how much data are typically available for a given Android user; (3) whether GLH recording rates depended on cell signal, and (4) whether GLH location points are spatially accurate compared to established GPS tracking units. To address these questions, we collected GLH data among Android users and administered a survey addressing recent international movement, use of Google services, and technology use among individuals recruited through the University of Southampton in the United Kingdom. Among a subsample of these participants, we further validated the feasibility and accuracy of the GLH data by comparing GLH data to GPS data, and by correlating points recorded by the GPS and Android phone. Finally, we independently administered Google Surveys to Android users in several countries to address the proportion of users that have GLH data across high and middle-income countries.

## Methods

### Data collection

For the GLH and survey data collection, we recruited 25 individuals throughout the University of Southampton (ethics approval ERGO ID 23647) from October to December 2016, targeting people who use an Android device as their primary mobile device. After administering informed consent, participants were randomly assigned to one of two possible study groups: “GLH only” or “+GPS”. The “GLH only” group involved a single study visit where participants accessed and downloaded their GLH data and completed a self-administered survey. The survey included questions about phone model and Android version installed, past and present use of GLH and other Google services, opinions on data privacy, recent self-reported international travel, and health related questions. For those randomized to the “+GPS” group, the initial study visit consisted of the same process, in addition to carrying a GPS logger unit (i-gotU model GT-600) for the following 7 days. Technical details and validation of the i-gotU GPS unit are outlined elsewhere [28]. After one week, participants returned for a final study visit, where they returned the GPS logger unit and downloaded their GLH data again, providing GLH data for the 7 days corresponding to GPS tracker carriage. Study design is outlined in more detail in Additional file 1, including the GLH data download process and questionnaire.

We measured how much GPS and GLH data we obtained from each user, quantifying temporal and spatial extent of data and recording rates. We associated these measures with survey data to determine if data availability depended on technical details such as phone model and the version of Android installed. We also examined the correlation between data availability/breadth and a user’s utilisation of various Google services and data privacy perceptions more generally.

### Google surveys

To address the likelihood of Android users having GLH data across different countries, we administered online Google Surveys in Brazil, the USA, the UK, Japan, and Mexico to 250 Android users in each country (1250 total). These surveys are administered to users through the Google Opinion Rewards app. This service provides nationally population-representative results to researchers using weights based on self-reported age and gender, and location based on browsing history and IP address. Further details on the Google Survey weighting methodology can be found at https://www.google.com/analytics/resources/whitepaper-how-google-surveys-works.html. In each of these surveys, we asked users if their Google account has GLH reporting enabled (“Yes”, “No”, or “Don’t Know”), instructing users that they are able to check under “Your Timeline” in the Google Maps app.

### Comparison with common types of mobility data

To better contextualize the temporal breadth and resolution of GLH data, we performed a rapid literature review in PubMed using the following search terms in the title/abstract: ‘human mobility’, ‘travel patterns’, ‘human movement’, ‘GPS tracker’, ‘Call Data Records’, ‘migration’, ‘population dynamics’ or ‘mobility networks’. This search resulted in 36,982 publications, which we further restricted to studies on humans published within the past 10 years, resulting in 2203 articles. Papers were selected for inclusion if they met the following criteria: (1) the study was published after 2008, (2) the study captured data on individual-level human mobility (i.e., social media check-ins, Call Data Records, GPS trackers, and travel history surveys), and (3) the study reported information on temporal resolution of analysis. We did not include review articles or studies modelling human movement using agent-based models or aggregate data, such as air traffic or commuter data. Some datasets had several associated articles (for example, CDRs provided for Senegal and the Ivory Coast through the D4D Challenge initiative); we therefore removed articles reporting on data previously included in the literature review. After reviewing article abstracts and methods, we identified a total of 43 suitable articles to include in our literature review [2, 6, 17, 22, 23, 28–65]. The table of studies used in this literature review is provided as Additional file 2.

### Cell tower comparison

To determine whether GLH recording rates depended on cell coverage, we quantified the relationship between rate of GLH data recording and distance from the nearest cell tower using a generalized linear model, including a randomly varying individual-level intercept to control for individual-level differences in ping rate. We obtained cell tower locations from OpenCellID.org, which synthesizes cell tower locations inferred from various smartphone apps and donated by mobile operators to build a database on cell towers throughout the world. This database was used previously to map hospital catchment areas [66]. Because Android devices occasionally stopped recording location history points for long periods, we restricted these analyses to points where the time between the last point collected was 1 week or less and to points within the United Kingdom, yielding a total sample size of 1,821,728 data points. We restricted the analysis to 1 week or less to account for very long periods when users may have either disabled the internal GPS functionality on their phone, or switched to a phone without an internal GPS, removing 43 data points in total.

### GPS validation

To validate whether the GLH data are as spatially accurate and frequently-collected as established GPS tracker data, we compared ping rates between users with both GLH and GPS tracker data, distance between recorded location points, and other metrics to address whether the GLH data were accurate and representative of overall movement. Specifically, we calculated the distance between GPS and GLH points for all minutes where both GPS and GLH data were recorded. If multiple coordinates were recorded in a given minute, we assigned the mean latitude and longitude for that minute.

We also aggregated both datasets to gridded surfaces of varying resolution (ranging from grid squares of 100 m by 100 m to 2500 m by 2500 m) and determined if GLH and GPS points were recorded within the same grid squares for each hour. We used gridded surfaces because researchers often combine location data with gridded spatial data that informs the risk of interest, such as malaria prevalence [67], healthcare accessibility [9], or air pollution [68]. We calculated percentage agreement for each hour by dividing the number of grid squares with points in both datasets by the total number of grid squares with points across both datasets. For each hour, if $C_{GLH} \cap C_{GPS}$ is the number of grid squares with points in both the GPS and GLH data and $C_{GLH} \cup C_{GPS}$ is the number of grid squares with points from either dataset, then the percent agreement $a$ for that hour is $a = \frac{{C_{GLH} \cap C_{GPS} }}{{C_{GLH} \cup C_{GPS} }}$. Therefore, if all the grid squares with GLH points also had GPS points and vice versa for a given hour, we recorded 100% agreement for that hour at that gridded surface resolution.

We repeated this analysis after interpolating linearly between points for minutes where no data were recorded. Linear interpolation is commonly used to fill in location information [69, 70], as GPS tracker data often have large gaps with no data recorded, particularly when the device is not moving, which we also observed in the GLH data.

We also determined if one dataset captured more travel than the other during the week that the GPS trackers were carried, by comparing the numbers of trips away from the previous night’s residence recorded in each dataset. We accomplished this by assigning a residence using the last location point from the GPS tracker data from the previous night. This assumes that the GPS trackers provided an accurate location for where that person spent the night, and we then calculated numbers of trips in the GPS and GLH data by counting the number of times people more than 100 m away from their daily assigned residence. Here, 100 m was chosen to define travel away from home due to the apparent accuracy of the GLH data compared to the GPS tracker data. We compared these using different definitions of trips away from home, ranging from at least 10 min away from home to two hours.

## Results

### GLH data

Among the 25 participants in our pilot study, two individuals reported that their GLH was disabled. A further two participants had no GLH data, suggesting they thought GLH recording was enabled, but was disabled in reality. This resulted in GLH data from a total of 21 participants, or approximately 85% of our sample. Among all participants, 20% (n = 5) reported that they had ever disabled GLH services, while a further 28% (n = 7) reported not knowing if they had ever disabled it. Among those who had previously disabled the service, two reported doing so for privacy reasons, two reported not feeling the need to enable it, and one reported disabling it to save battery life. Two participants further reported turning GLH services back on specifically to utilise the Google Maps feature.

Our sample included a variety of Android phone models, with a plurality (n = 9) of respondents owning a Samsung Galaxy device. Other models included Huawei, Lenovo, Tecno, Infinix, Medion, Xiaomi, Asus, LG Nexus, Motorola, Blue Diamond, and OnePlus phones. The current Android operating system version on these phones varied between versions 4.4.2 through 7.0, and we found no significant difference in ping rate over the last three months of data collection with different Android versions or with different phone models (Additional file 3).

**Fig. 2:**
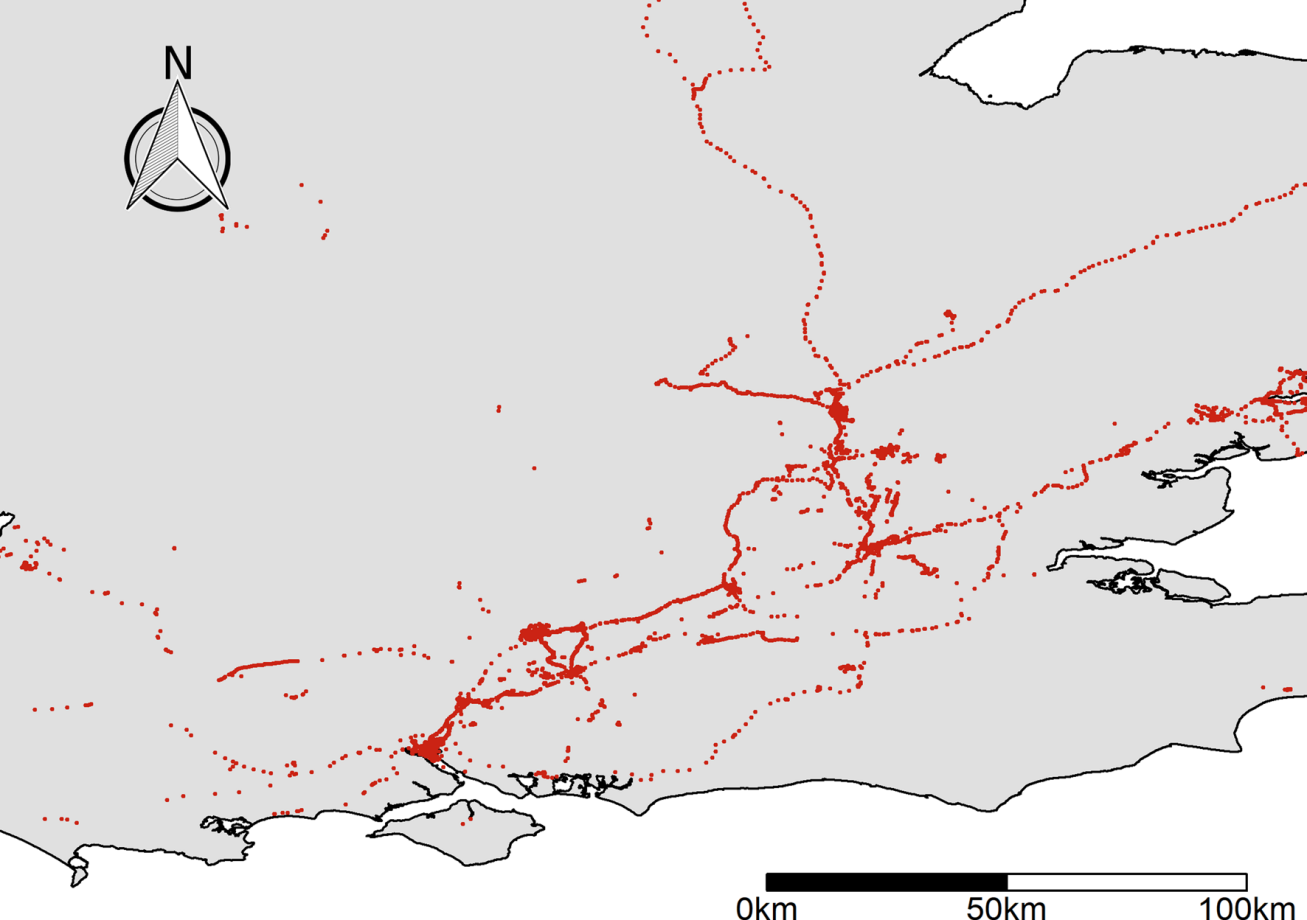
Aggregate GLH data (4.32 million points from June 2013 to December 2016) collected from study participants (n = 21). This map shows tracks across southern England

For the 21 participants with GLH data, we obtained a mean 205,000 location history points per user across an average of 367 days, yielding 4.32 million total geographic coordinates (Fig. 2). This often included days without any recorded data. On average, the beginning and end dates of location history points were 556 days apart, suggesting that phones did not record data during roughly 1/3 of days. This may be due to study participants not using an Android smartphone for the entire period, or due to study participants turning off location history collection or the GPS service on their smartphone. The actual proportion of days with no data ranged from 0% to 90% across the 21 users, which did not appear to correlate with Android version or phone model (Additional file 3) but did negatively correlate strongly with total number of points collected, suggesting no-data days were due to other factors.

We identified numerous occasions of international travel, with locations recorded in 41 different countries across the 21 individuals. In the questionnaire, we asked participants the last country they visited outside of the UK, and 17 users reported traveling internationally in the past year. After excluding very short periods recorded in other countries (less than one day), the GLH data accurately captured the last visited country for 14 out of these 17 users. We excluded travel to a country for less than one day, as that likely indicates stopovers and would not typically be counted as international travel. For the three cases where GLH data did not capture the last country visited, two participants reported disabling data/GPS regularly.

**Fig. 3:**
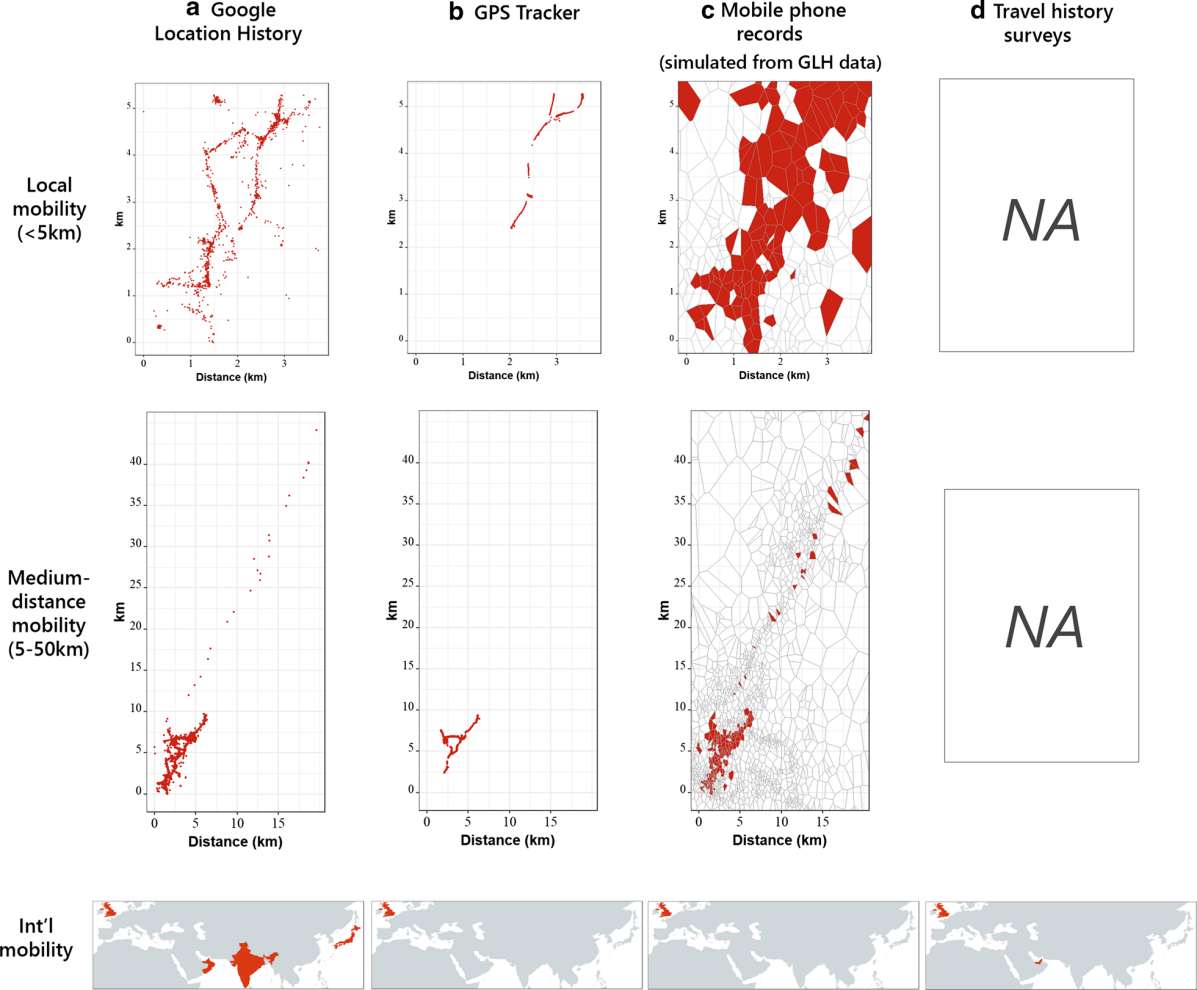
Location information available at different spatial scales from the **a** GLH, **b** GPS, **c** simulated mobile phone data, and **d** survey data collected during this study. **c** Mobile phone data shown here were simulated using the GLH data, assuming each GLH location point was a call or text event routed through the nearest cell tower. In the simulated mobile phone data, polygons represent Voronoi polygons drawn around cell towers from the OpenCellID dataset, and are colored red if any simulated call/text events were routed through the associated tower

Figure 3 shows GLH and GPS tracker data for a randomly chosen subset of individuals from the +GPS group, and differences in data collected at various spatial scales between the GLH data and the GPS trackers. This figure also shows simulated mobile phone (CDR) data, assuming each GLH location point corresponded with a call or text event, and using the OpenCellID dataset to inform cell tower locations, yielding Voronoi polygons around cell towers roughly 242.8 m^2^ in size on average after isolating the OpenCellID dataset to the mobile operator with the most towers. As location point recording occurred often every minute or more frequently during travel, this is likely a very large overestimation of call and text rates. Because CDR data generally only include calls and texts within networks that do not cross national borders, we excluded any international travel from the simulated CDR data. This figure also includes the countries reported as visited during the in-person questionnaire.

Notably, the GLH data recorded 41 international trips across 21 individuals (excluding countries where the person spent less than one day, to account for stopovers during travel), while the GPS data captured zero international trips for six individuals in the +GPS group due to the short duration covered, and the travel history data captured 18 international trips due to the questionnaire recording the most recent country visited in the past year. When comparing numbers of trips recorded during the week when the +GPS group carried GPS trackers, we found similar trips in both datasets regardless of the minimum amount of time away required to count a trip. Specifically, for the six individuals where we compared this analysis, if the minimum duration to qualify as a trip was 10 min away from home, the mean number of trips identified was 10 (minimum 6, maximum 15) in the GPS data, and 10 (minimum 7, maximum 15) in the GLH data. If the duration was set to 120 min, the GPS data recorded 7.2 trips (minimum 5, maximum 10), while the GLH data recorded 7.4 trips (minimum 4, maximum 10).

### Google surveys

Among 1250 Android users, most countries had the highest proportion of users reporting having GLH reporting enabled, ranging from 43% in Japan to 72% in Mexico. In comparison, the proportion of users reporting having GLH reporting disabled (as measured by a ‘No’ response to the question) ranged from 5.6% in Brazil to 17.5% in the UK. Other users reported not knowing whether this feature is enabled, ranging from 20% in Mexico to 51% in Japan. Additional file 3 includes more detail on these survey results.

### Comparison with common types of mobility data

**Fig. 4:**
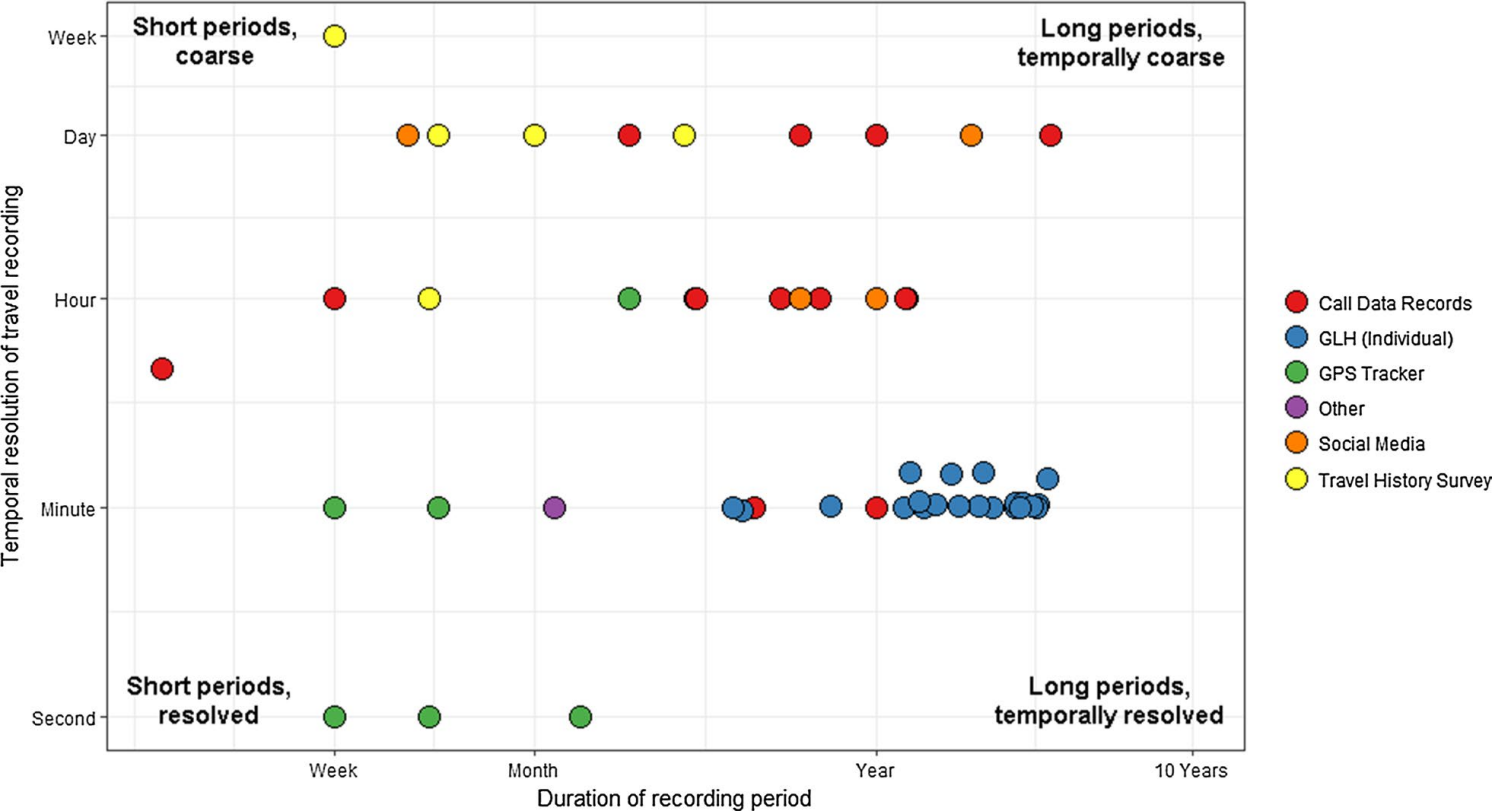
Temporal breadth and resolution of various data types, from studies found through a rapid literature review. The temporal breadth is the period of time over which travel was reported for that study, and the resolution is the greatest accuracy in mobility (i.e. for CDR and GPS data, the average frequency that location points were recorded, while for travel history surveys, the minimum trip duration for a trip to be recorded). GLH points (in blue) represent individuals in our study, to illustrate the range of breadth and resolution of the collected data

Figure 4 visualizes the temporal resolution and duration of travel period by data type, with the GLH data collected during this study included. We found that generally, GPS tracker data captured trips at the highest temporal resolution, while travel history surveys did not often capture shorter-term (less than 1 day) travel, and social media and call detail records enabled by new technologies had the longest travel periods recorded, frequently spanning many months or years. We also found that the GLH data fill a unique niche spanning travel periods of many years similar to CDRs, while also having high temporal resolution similar to GPS tracker data.

### Cell tower comparison

We found a statistically significant positive relationship between time since the last GLH data point and distance from the nearest cell tower (*p* < .0001) in a generalized mixed model that included user ID as a random effect to account for individual-level differences in recording behaviour. In this model, we only included points where the time since the previous recording point was less than 1 week, to account for participants potentially using a phone without GPS functionality or disabling their Android phone’s internal GPS. Overall, GLH recording rate increased by 1 s for every additional 7.5 m from the nearest cell tower (regression coefficient .1325). This relationship appeared to be partly driven by high recording rates (every 30 s or less) less than 1 km from the nearest cell tower. When repeated using only points separated by 30 s or more, this relationship became a non-significant positive trend between cell tower distance and ping rate (*p* = .2721). Additional file 3: Fig. S5 shows the relationship between time since last recorded point and distance from the nearest cell tower in more detail.

### GPS validation

To validate GLH data as compared to established methods such as GPS trackers, we quantified the spatial percent agreement of GLH and GPS data points. In total, there were 1267 min where both GPS and GLH data were recorded. For these minutes, the GLH data were typically less than 100 m away from the GPS data in the corresponding minute, with a median distance of 64 m separating the GLH and GPS data.

**Fig. 5:**
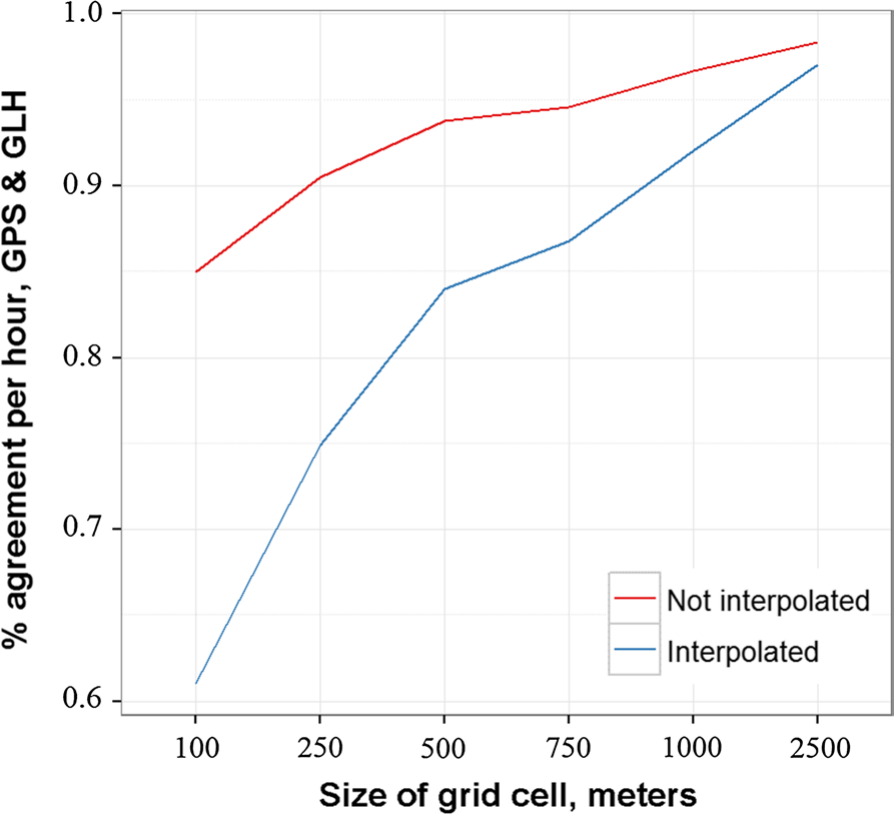
Agreement in grid cells visited in GLH and GPS datasets across 7 individuals, for varying grid cell sizes. Only hours with both GPS and GLH data were used. Interpolated refers to linearly interpolating locations for minutes with no data

We compared percentage agreement across varying grid cell sizes, which helps identify the spatial resolutions at which GLH data are functionally equivalent to GPS tracker data. We found that the two datasets had roughly 85% agreement when using a gridded surface of cells that were 100 × 100 m. As expected, this percentage increased with larger grid cells (Fig. 5). The linearly interpolated data generally agreed less, with only 60% agreement using a gridded surface of 100 m × 100 m cells. At 500 m × 500 m, the interpolated data began to agree similarly to the non-interpolated data, with roughly 85% agreement between the two datasets.

Two individuals in the GPS + group contained days both with and entirely without GLH data collection, critically allowing us to examine travel patterns on days without GLH data, thereby making inferences about whether these data are not collected as a function of movement. Importantly, the qualitative patterns as measured by the GPS tracker in days with and without GLH data did not appear to differ for these individuals. Specifically, the radius of gyration, a common aggregate measure of movement [2], was .586 decimal-degrees during days without GLH data versus .677 during days with GLH data, suggesting that gaps in the GLH data may not depend on mobility, and are due to user behaviour or other non-mobility related factors.

## Discussion

Our results suggest that GLH data could provide unmatched individualized human movement information and address key gaps in currently-available data, including many trips over long periods of time while being spatially resolved (Fig. 3). These data are functionally similar to GPS tracker data (Figs. 3, 4), but are easier to collect in a survey-based study than GPS data and less prone to participant usage issues, as they are passively collected and are easily retrieved by users. We collected these data in conjunction with a questionnaire that addressed self-reported international movement patterns, use of Android phones and various Google services, and provide our study materials for further use in Additional file 3. Other surveys may similarly collect broad demographic information to link with GLH movement data, which currently represents an important gap in human mobility research.

We found that GLH data can provide mobility data over periods and at a resolution infeasible from other typical sources of movement information (Figs. 3, 4) [15, 71], and were more temporally resolved and broad than data used in most recent studies (Fig. 4). We collected roughly two years of data on average from study participants, while studies using GPS trackers generally are only able to collect 1–2 weeks of location data at a time due to battery life issues [28]. Because the GLH data covered much longer periods, we were able to identify not only very short-distance, circulatory movements (top, Fig. 3a), but also numerous international trips (bottom, Fig. 3a). Furthermore, GLH data contain more fine-scale information than CDR data, since CDR data only identify the cell tower used (top, Fig. 3c), and in this case, cell towers covered an area of 242.8 m^2^, suggesting lower accuracy than the GLH data. In reality, CDR data provide less location information than Fig. 3 implies, as calls and texts occur typically much less frequently than the GLH recording average of once per minute, and towers are typically less densely placed than in urban centers like Southampton. On larger spatial scales (bottom, Fig. 3), the GLH data recorded more information than could be reasonably expected to be collected through travel history surveys, collecting information on travel to up to countries, where travel history surveys are generally treated as unreliable after the first few recollected locations. Importantly, the GPS tracker data recorded no international mobility due to the short time span of data collection, and CDR data generally do not include international movement due roaming on cell networks in other countries.

The GLH data were as accurate and representative as GPS tracker data from the same period if aggregated to an appropriate temporal and spatial resolution, such as 500 m or greater (Fig. 5; Additional file 3: Fig. S3). Even still, we found recorded GLH points were generally within 100 m of the corresponding recorded GPS data point, which is significantly better than the best-case scenario of 250 m found with the CDR data in Southampton (Fig. 3). Across a weeklong timescale, these data also generally strongly agreed both when interpolated between minutes and when non-interpolated on grids of 500 m × 500 m or coarser. These are conservative estimates as they assume the GPS tracker data were perfectly accurate, where GPS tracker points are known to vary up to 20 m even when the GPS unit is stationary [28]. While we did observe gaps in GLH data collection, these gaps did not appear to correlate with movement in the two individuals where gaps occurred during GPS data collection and therefore allowed for location tracking when no GLH points were recorded. GLH data collection did appear to correlate with distance from the nearest cell tower, but found that this source of bias can be mitigated by aggregating location points to each minute or longer.

### Broad applications

Understanding how people move throughout their daily activities within the context of spatial risks will be important for the health and social sciences, as this would enable a better understanding of the environmental drivers of chronic disease, socioeconomic inequalities, and other issues that involve long-term differences in exposure and mobility. GLH data could yield important insights into disparities in health, wealth, and wellbeing in settings where these analyses were previously impossible, such as in urban centres when considering risks associated with long-term exposure. Because these data are opt-out and are passively-collected as an Android user carries their smartphone, they will often include locational information over longer periods than it is possible to obtain from other sources that collect data at a similar spatial resolution (Fig. 3). While wealthier urban populations tend to have better access to resources such as green spaces [72] and high quality food [73, 74], nearby poorer populations often experience worse social outcomes due in part to the effective inaccessibility of such resources, and use of these resources is best measured across long periods. In these settings, small distances separate populations that spend time in very different places, but GPS trackers generally cannot cover the periods needed. The high resolution of GLH data mean they are one of few viable sources of information for better understanding and mapping these differences towards mapping activity spaces and travel routes across long periods (Fig. 2, 4). These inferences can assist infrastructure and intervention planning, as identifying routes used to access various social and health-oriented resources could identify the most important routes for ensuring equitable infrastructure access [75]. By providing urban planners with better context on not only which infrastructure is most used, but which populations are using various resources, could help promote socially sustainable transport [76], and could help inform urban planning in the context of historically socially-isolated communities [77].

The directly collected nature of obtaining a user’s GLH data also means the data pair well with other useful information such as demographics and health related outcomes. As fine scale mobility can differ greatly between people based on income, gender, and other sociodemographic factors, survey data combined with GLH data could determine whether important travel patterns depend on socioeconomic factors, to help target and account for vulnerable populations. Due to their uniquely identifiable nature, however, linking sociodemographic and health information with high resolution mobility data such as GLH raises important privacy considerations, necessitating an ethical obligation to protect participant confidentiality. Confidentiality of sensitive geographic data has been similarly faced by household survey programmes such as the Demographic and Health Surveys (DHS) who release publicly available georeferenced data. Towards this, the DHS outlines common practices in ensuring participant confidentiality, using established techniques such as aggregate data disclosure and geographic masking techniques such as displacement [78]. By employing these measures, researchers may ensure the benefits of their study do not outweigh individual risk of identification.

### Limitations

Critically, GLH data can only be obtained by the user, necessitating a study design similar to typical survey-based research and similar sample sizes. Future work could facilitate faster data collection, by providing an automated process for participants to easily view, download and provide their GLH data to researchers. While this requirement increases the cost of studies that collect GLH data, actively engaging participants during data download also permits simultaneous collection of other demographic or health related information, such as recent infection status of various diseases.

Though the active nature of data retrieval makes large sample sizes difficult to obtain, this makes GLH data complementary with CDRs where both are available. Where GLH data provide fine-scale and international travel and can be collected with individual-level socioeconomic data, CDR data provide comprehensive travel patterns for all people across a country but do not include international movement or locations between call and text events. The two could be directly linked by recording phone numbers when collecting GLH data and linking individuals with their corresponding CDR data. In lieu of directly linked data, relationships between risk, socioeconomic status, location, and mobility in GLH data could help predict risk or socioeconomic characteristics for individuals in CDR data.

We enrolled study participants using non-representative recruitment methods, potentially biasing participants towards those more engaged with new smartphone technologies. This may therefore result in an overrepresentation of GLH data than would be expected in other settings. Further, our study population is comprised of residents within the United Kingdom, which may be more likely to own smartphones and frequently use app-based services such as Google Location History. We confirm that Android users are likely to have GLH data in a variety of countries using Google Surveys (Additional file 3: Fig. S1), but future work will need to better describe smartphone-owning populations and quantify how long various populations are likely to have owned smartphones in areas where GLH data may be collected.

Along these lines, GLH data are currently impossible to collect for many populations, as data collection requires that populations have Android smartphones, and have reliable mobile infrastructure and Internet connection for data retrieval. While these data will likely not be relevant for some of the most vulnerable populations in low income settings, Android phone use is increasing globally and becoming available to more people each year [21, 26]. In many middle income countries, Android has surpassed Windows and all other operating systems as the most common OS for accessing the Internet, and in many of these countries, people are opting to use mobile phone primarily as computing devices over desktop or laptop computers [26].

It is also possible that Android users do not have GLH data, most likely due to having GLH data reporting disabled. In our Southampton sample and in our Google Survey results, we found that this likely does not affect data collection, as a majority of Android users reported having GLH reporting enabled in all countries but Japan in our Google Survey results. Across these surveys, typically 10% or less reported having GLH reporting disabled (Additional file 3: Fig S1). While 20–51% of respondents did not know whether GLH reporting was enabled, because GLH reporting is opt-out, it is likely most of these users have it enabled.

Ultimately, GLH data are a greatly underutilized and novel dataset for understanding human movement, and for mapping activity spaces. While there is a strong bias in populations with GLH data to be wealthier than those without, Android phones are becoming the first and only device purchased to access the Internet and various web services in many middle and lower income settings, making these data increasingly appropriate for a wide range of scientific questions.

## Additional files

**Additional file 1.** Study materials.

**Additional file 2.** Literature review data.

**Additional file 3.** Google Surveys and other Google Location History data (GLH) analysis.
