# Supplementary material for: Using Google Location History data to quantify fine-scale human mobility
Source: Int J Health Geogr. 2018 Jul 27;17:28. doi: 10.1186/s12942-018-0150-z (PMC6062973; doi:10.1186/s12942-018-0150-z)
Supplement: Supplementary file 1 — Additional file 1. Study materials. [file 12942_2018_150_MOESM1_ESM.docx]

Additional file 1

Study materials

**Study questionnaire**

Inclusion criteria:

● 18 or older

● UK resident

● Currently uses Android phone as primary mobile device

Screener Questions:

1) Do you use an Android phone as your primary mobile device?

a) Yes

b) No

c) Don’t know

Missing Demographics

1) Do you currently have Google Location history enabled? (provide instructions on how to check)

2) Have you ever disabled Google Location history?

a) If so, why did you choose to disable Google location services?

b) If applicable, why did you choose to turn Google location services back on?

Core Questionnaire:

1) What is the model of your current Android-enabled phone? (Provide instructions how to check)

2) What is the version of your current Android operating system? (Provide instructions how to check)

3) For approximately how long have you owned any Android-enabled smartphone?

a) <1 yr

b) 1 - 2 yrs

c) >2 yrs

d) Don’t know

4) How much do you agree or disagree with the following statement? I am worried about how much data companies have about me on the internet.

a) Definitely disagree

b) Tend to disagree

c) Tend to agree

d) Definitely agree

5) How often do you use the following Google services?

a) Gmail

■ Very often (more than once a day)

■ Often (more than once a week)

■ Occasionally (less than once a week)

■ Rarely (less than once a month)

■ Not at all

b) Google Maps

c) Google Docs

d) Google Drive

e) Google Calendar

f) Google Plus/Hangouts

g) Google Wallet

h) Other Google services (please specify)

Location Questions:

1) How many international trips have you made in the past year?

a) 0

b) 1 to 3

c) Greater than 3

2) If you have traveled internationally in the last year, what country was your most recent destination? (list countries)

a) What month did this travel occur?

3) How often did you use your Android-enabled device during your most recent international travel?

a) Every day

b) More days than not

c) Occasionally

d) Rarely

e) Not at all

Health Questionnaire:

1) How would you rate your health?

a) Excellent

b) Very good

c) Good

d) Fair

e) Poor

2) How many times have you visited a hospital in the past 12 months?

a) 0 times

b) 1 - 2 times

c) 3 - 5 times

d) 6 + times

e) Don’t Know

**Instructions provided for data download**

1. Go to <https://www.google.com/settings/takeout> and log in using the Google account associated with your Android phone.
2. Click “Select none” under “Select data to include”.
3. Scroll down to “Location History”, and select only that, and make sure it indicates “JSON format”.
4. Click “Next” at the bottom, and click “Create archive” using the defaults (.zip file, sending download link to email).

<<<Survey administered at this point>>>

1. Go to gmail.com using the account associated with your Android phone, you should receive an email titled “Your Google data archive is ready”.
2. Click “Download archive” within the email and click “Download” after logging in with your Google account.
3. Open the zip file, open the “Takeout” folder and the “Location History” subfolder, and unzip **only** the file LocationHistory.json.
4. Place LocationHistory.json into the folder on the laptop for storage
