# Supplementary material for: Using Google Location History data to quantify fine-scale human mobility
Source: Int J Health Geogr. 2018 Jul 27;17:28. doi: 10.1186/s12942-018-0150-z (PMC6062973; doi:10.1186/s12942-018-0150-z)
Supplement: Supplementary file 3 — Additional file 3. Google Surveys and other Google Location History data (GLH) analysis. [file 12942_2018_150_MOESM3_ESM.docx]

Additional file 3: Google Surveys and other Google Location History data (GLH) analysis

1.1 Google Surveys

In five countries (the UK, the USA, Japan, Mexico, and Brazil), we asked 250 Android users per country through a Google Survey whether they had Location History reporting enabled. This survey notifies potential respondents of the survey on their Android smartphone, and is administered through the Google Opinion Rewards application. Because these surveys are administered entirely through the Android smartphone, it is an especially useful tool for quantifying GLH data recording patterns. The 250 user sample size was selected based on the number needed to achieve significance at a 95% confidence level given 10% of users reporting having it disabled. We obtained this original 10% value from an initial survey of 50 Android users in Brazil.


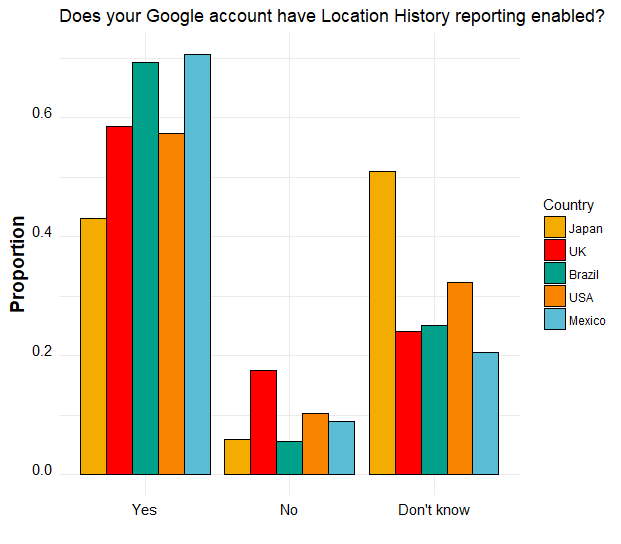


***Fig S1.*** ***Proportion of Google Survey respondents reporting having Google Location History reporting enabled*** *(n = 1250). 250 Android smartphone users were surveyed in each of Japan, the UK, Brazil, the USA, and Mexico. Results are weighted by gender, age, and first level administrative unit of residence.*

1.2. GLH data analysis

Across the 21 study participants, the GLH dataset included numerous occasions of international travel, with locations recorded in 41 different countries across the 21 individuals (Fig S2).


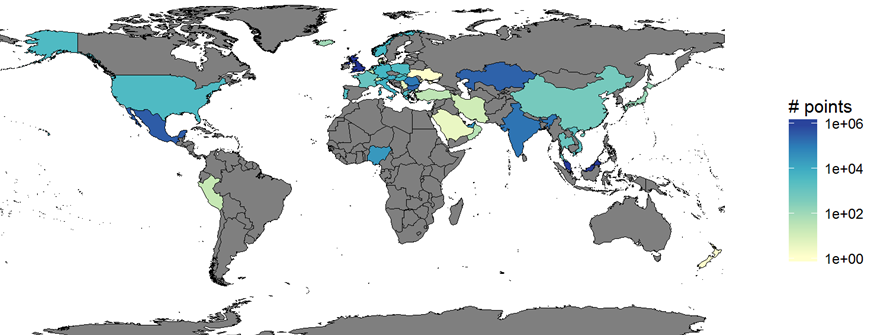


***Figure S2. Numbers of points recorded in each country across all 21 individuals with Google Location History data.*** *Plot includes 4,326,762 total points.*

Figure S3 shows the distance between GLH and GPS points in minutes where both recorded coordinates, which occurred in 1267 minutes across all 21 study participants. Where multiple points were recorded in the same minute in either data set, we used the mean latitude and longitude for that minute.


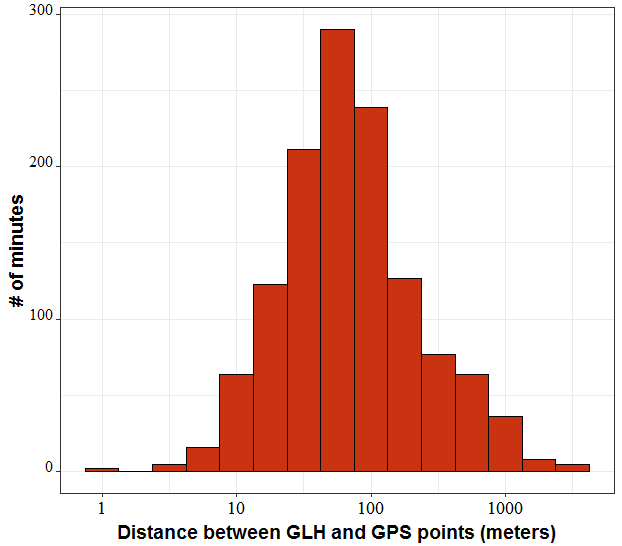


***Figure S3. Distance between GLH and GPS points, for minutes where both were recorded.***

Figure S4 shows the number of coordinates recorded across each user, with a median of 194,335 points recorded.

*
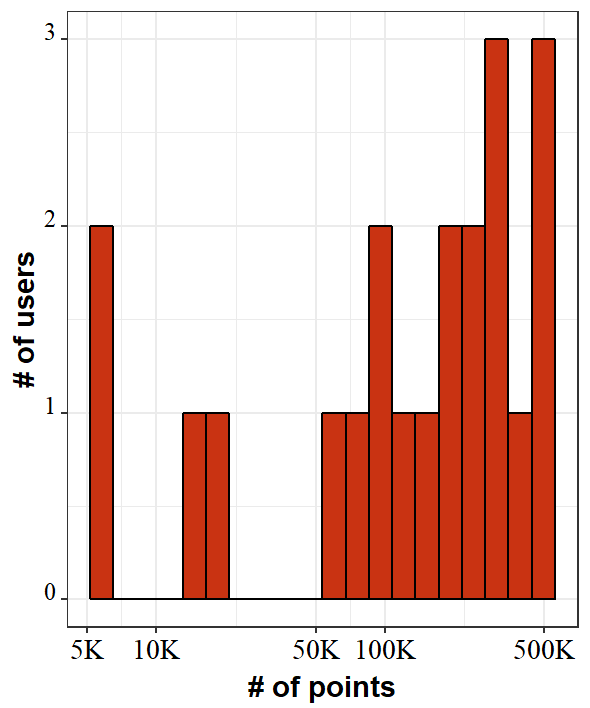
*

***Figure S4. Number of points recorded per user, across 21 users with GLH data.***

*
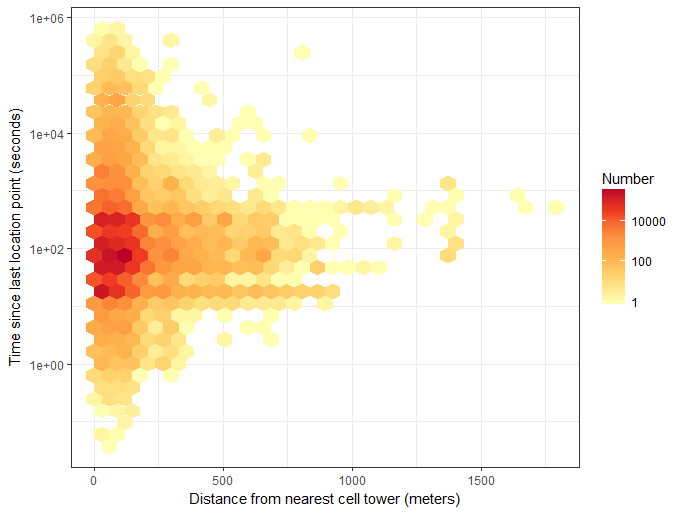
*

***Figure S5. Relationship between distance from nearest cell tower and time since last recorded point.***
